# Validation of the Maltese Adaptive Auditory Speech Test (AAST)

**DOI:** 10.3390/audiolres12040037

**Published:** 2022-06-26

**Authors:** Pauline Miggiani, Frans Coninx, Karolin Schaefer

**Affiliations:** 1 Audiology Clinic, Mater Dei Hopsital, MSD 2090 Msida, Malta; paulinemig@gmail.com; 2 IfAP, Institut für Audiopädagogik, 42697 Solingen, Germany; f.coninx@ifap.info; 3 Department of Special Education and Rehabilitation, Faculty of Health Sciences, University of Cologne, 50931 Cologne, Germany

**Keywords:** adaptive speech test, speech audiometry, pediatric speech test, speech in noise test, Maltese

## Abstract

The Adaptive Auditory Speech Test (AAST) was developed to record the Speech Recognition Threshold (SRT) in children in quiet or with background noise. AAST is an interlingually valid and reliable standardised tool with speech material developed in several languages. The Maltese version of the Adaptive Auditory Speech Test (AAST) was developed to examine the speech recognition skills of 208 children and 40 Maltese-speaking adults in quiet, noise and high frequency. The aims were to determine the norms in these three settings in adults and children aged 4 years and older. The Maltese version of AAST confirms an age dependent norm threshold with a significant improvement in threshold being observed as children grow older, similar to other AAST versions. This was evident across the three test settings. An approximate difference of 10 dB was also noted between 4-year-old and 10-year-old children in AAST in quiet. Thresholds of 10-year-olds and adults were similar in both the quiet and high frequency versions. Implications for post Universal Newborn Hearing Screening using these tools are addressed.

## 1. Introduction

Speech audiometry is an integral part of an audiological test battery. It is used alongside pure-tone audiometry to quantify a person’s ability to hear and understand speech clinically. A major component of speech audiometry includes the measurement of the hearing threshold for speech. The speech recognition threshold (SRT) test is defined as the lowest level at which the individual can correctly recognise 50% of words presented [1]. Speech audiometry allows for quantitative measures of speech understanding through test materials that closely resemble everyday listening tasks. In clinical practice, it provides a means to cross-check the validity of the pure–tone (also known as air conduction) audiometry results. Speech audiometry adds diagnostic and prognostic value and also supports rehabilitation and treatment decisions in relation to hearing aids and cochlear implants [2].

Currently, the clinical assessment of speech intelligibility commonly includes testing both in quiet and in noise since scores cannot be predicted based on an audiogram, and word recognition in quiet does not predict scores in noise [3]. Testing in noise approximates a more realistic environment and helps to avoid ceiling effects, which are more common when testing in quiet. This is also recommended by professional audiology organisations [4]. Speech-in-noise testing is useful in the selection of amplification devices, the determination of patient expectations and as an outcome measure in the management of hearing loss [5,6]. Speech-in-noise tests can be classified as either fixed or adaptive. In fixed tests, the signal-to-noise ratio (SNR) is fixed, as the speech signal is presented at one level, and the noise is presented at another level. The result is displayed as percentage correct, making it easy to deliver the results to the patient. In an adaptive test, testing is conducted to approximate the SNR needed for 50% speech recognition. Either the speech or noise level is adaptively changed whilst the other is fixed. For example, the QuickSin test [7] fixes the speech level and adaptively changes the noise level. The Hearing in Noise Test [8], on the other hand, adaptively changes the speech level while keeping the noise level fixed. However, both tests converge to find a SNR value where a SRT of 50% is reached. This will help in identifying the 50% correct point quickly and reliably as it does not require as many test items for obtaining the final score. Adaptive tests also help to avoid ceiling effects. Everyday listening commonly occurs in the context of acoustic challenges that de-grade speech. When individuals hear, they must match the rapid incoming acoustic signals to their stored representations of words in order to extract the intended meaning. In speech audiometry, the number of words recognised correctly is an important measure, but so is the concept of listening effort. This applies even when speech recognition scores are high. The British Society of Audiology proposed the following definition in a white paper: “the mental exertion required to attend to, and understand, an auditory message” [9]. According to the Ease of Language Understanding Model [10], listening to speech is rather effortless in ideal listening conditions. Speech recognition may require more effort when the quality of the speech signal is degraded due to noise, hearing loss or complex language.

To measure this cognitive load during listening, accuracy is recorded by the percentage of correctly identified words, whilst speed is assessed through response time. The concept of response time as a measure of cognitive load in speech recognition studies shows that as listening conditions become more degraded, listening effort also increases as evidenced by a response time increase [11,12]. Besides behavioural measures such as response time, self-rating scales and physiological measures are also used in research to measure listening effort [13].

### 1.1. Speech Audiometry in Children

Audiologists are faced with the challenge of choosing the most appropriate hearing test to administer to a given child. Pure-Tone Audiometry (PTA), in combination with tympanometry, is the gold standard in children above 4 or 5 years of age in identifying hearing loss and middle-ear disorders [14]. Over the years, the importance of incorporating speech audiometry into the test battery has increased [15,16]. Tests focusing on speech understanding provide relevant information about the auditory system and make it possible to predict the development of different skills in children, such as language, reading or cognitive abilities [17].

Several variables may affect performance in the paediatric population. Language skills, vocabulary, age and cognition may potentially impact the results. A test’s characteristics may also have an influence. The type of stimulus and response used, the type of reinforcement, if any, and the memory load can affect a child’s performance on a test [18]. Kosky and Boothroyd [19] recommend that paediatric speech tests should be motivating; age appropriate in terms of attention, cognition and fine motor skills; independent of speech production skills, vocabulary and higher language skills and able to assess ability to communicate in daily life. The aim is for speech recognition to be assessed using valid and reliable clinical tests. Open-set tests involve the child repeating back a stimulus word verbally, forcing the child to retrieve the item from all possible words in their lexical memory. In closed-set tasks, the child selects a word from a restricted set of responses (usually pictures), thus limiting the number of comparisons the child needs to carry out. Clopper et al.’s study [20] confirms that word retrieval plays a larger role in open-set tasks. Forced choice picture pointing tasks are thus frequently used to evaluate speech recognition in young children since they do not rely on the child’s speech production skills or accurate scoring of the tester. Word picture tests also involve less cognitive skills than sentence testing. Whilst sentences may provide an assessment of daily conversation, they may offer additional cues, which might result in better scores.

### 1.2. Adaptive Auditory Speech Test (AAST)

AAST was developed by Coninx in 2005 in order to record the Speech Recognition Threshold (SRT) easily, quickly and reliably in children in quiet or with background noise. Using a closed set of only six stimulus words, the AAST is minimally dependent on an individual’s lexicon. Since AAST is performed as a picture-pointing task via a touchscreen, the child points to the corresponding test item that is heard from the headphones (or speakers). Stimulus words are spondees or tri-syllable words. Since the AAST is an adaptive speech test, the response to the previous test word determines the level at which the next stimulus word is presented. If the response is correct, the intensity decreases, and if incorrect or there is no response, the intensity increases. The AAST stops automatically after a set number of wrong answers, and the SRT is automatically calculated by the AAST program. The average testing time is approximately one minute per test condition (quiet, noise). The tester has no role in the analysis other than comparing the SRT to the calculated norms in that language.

Speech material for the AAST is available in several languages, including German, English, Dutch, Arabic, Vietnamese, Spanish, Polish, Luxembourgish, Mandarin and Ghanaian.

Coninx [21,22] validated and normed the test on German children (4–12 years of age). He reported higher SRTs (approximately 10 dB) in 4-year-olds than 11-year-olds. Additionally, 8-year-olds in his study performed as well as adults. This could possibly be due to the developing phonological awareness and attention skills in the younger children. Based on these findings, Coninx suggested that age and SRT are interdependent. The psychometric curve for the AAST was 14%/dB for speech-in-noise measurement and was comparable to the Oldenburger Kinder Satztest [23], with slopes of 6–8%/dB in quiet and 12–14%/dB in noise. Other AAST versions report similar results. Offei [24] reported slope values of 10.2%/dB for the Ghanaian version and 8.2%/dB in quiet and 8.4%/dB for noise in the Vietnamese version [25].

Initially designed for 3–4-yearolds, it can also be used to test older children and adults alike [25,26]. Applications for the AAST also include the verification of aided thresholds with hearing aids and cochlear implants [27] and the screening of Auditory Processing Disorder [28].

In summary, the AAST is an interlingually valid and reliable standardised tool with several advantages, namely that it (1) is available in several languages, (2) has a short testing time, (3) is a closed-set task, suitable for young children, (4) has an interactive display for interactive assessment of the SRT, (5) is an adaptive test, preventing a ceiling effect, (6) can be carried out in quiet and in noise and (7) tests progress of hearing development in children with hearing aids and cochlear implants.

### 1.3. The Maltese Language

Maltese is the national language of the Maltese islands. Aside from Maltese, English is the only other official language of the country, with over 62% having a good command of it [29]. Most of the Maltese population can therefore be classified as bilingual. In general, Maltese is used at home and within the community, whilst English is used in higher educational contexts [30]. Most children residing in Malta can be classified as either simultaneous or sequential bilinguals. Whilst spoken English still carries a higher social status, the majority of children are exposed predominantly to Maltese and then to English [31]. Once children start school, they are simultaneously exposed to both languages [32].

The Maltese language originates from Arabic, but it is the only Semitic language which is officially written in the Latin alphabet [33]. It has continued to evolve through contact with romance languages such as Sicilian, Italian, and, later on, English. This uniqueness reflects the history of different rulers that once occupied the islands [30]. As with other Semitic languages, Maltese is rich in consonants. Maltese has 30 letters in its alphabet, six of which are unique to the Maltese language (ż /z/, ġ /dʒ/, ħ /h/, ċ /tʃ/, għ (mostly silent) and ie /i:/ [30]. Maltese phonology has a similar consonantal phonetic inventory to English. There are only two additional Maltese phonemes, /Ɂ/ and /ts/, while the English /θ/, /z/ and /ð/ phonemes are not part of the Maltese inventory [31].

Although speech audiometry materials have been developed in several languages, there are currently limited normed materials available in the Maltese language that would enable testing of speech perception skills, especially in children. Thus, the purpose of this study was to develop a Maltese version of the AAST that can be used to measure the SRT in children and adults whose native language is Maltese. Normative values were also developed from the data

## 2. Materials and Methods

### 2.1. Development of the Maltese AAST

The Adaptive Auditory Speech Test is a computer-based test that assesses the Speech Recognition Threshold. In most language versions, the speech material uses six spondees such as Eisbär (polar bear), Schneemann (snowman), Fussball (football), Flugzeug (airplane), Handschuhe (gloves) and Lenkrad (steering wheel) as speech stimuli [21]. The test subject must point to a picture to identify the word. The criteria for selection of the 6 AAST words for the Maltese version were as follows:

- 3–4-year-old children know the meaning of the words and recognise a picture of the words
- The words must have the same prosodic pattern: S-S (spondee), S-W-W (tri-syllable, first syllable stressed) or W-S-W (tri-syllable, second syllable stressed). (S = strong, W = weak).
- The words must be maximally different at the phoneme level. Preferably, the phoneme statistics should correspond to the frequency of occurrence in the language being adapted.

This means that the phonemes of all the 6 words must agree with the general distribution of consonants and vowels in the standard language. A search of the Maltese literature confirmed that there is no distribution curve for the Maltese language. The first chapter of a Maltese adult reading book was selected and used to develop the Maltese phoneme distribution curve. A total of 80 lines of text were phonetically transcribed, and the percentages for each class of phonemes (stops, fricatives, approximants, nasals/laterals, affricates/trills) were calculated.

Six words were then selected according to the above-mentioned criteria. The final words selected for the Maltese version of AAST are as follows: car (kɐrɔtsɐ), glasses (nʊtʃɐ:lɪ), snail (bebu:ʃʊ), presents (rɪgɐ:lɪ), key (tʃɐvɛttɐ) and monkey (ʃɐdɪ:nɐ).

**Figure 1:**
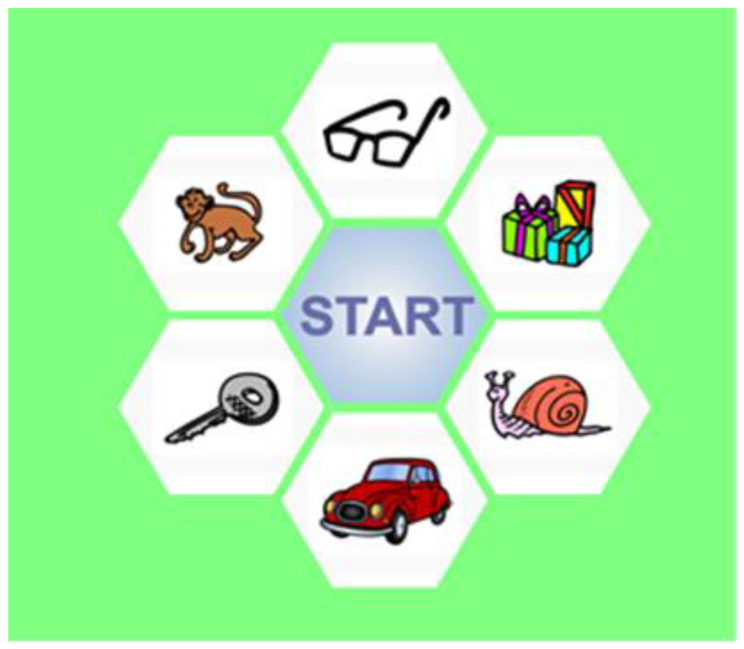
Interface of the pilot version of Maltese AAST.

Pictures for the Maltese AAST were based on the same 3 criteria used in other AAST versions [21,24,25]. They were all in the same style, colored and in JPG format (201 × 174 pixels). The pictures were piloted on 20 children, aged 3–4 years old. More than 95% of the children recognised all the pictures except for one. The picture was changed, and following retesting, the word was replaced. After retesting, all the pictures were deemed appropriate for the test. See Figure 1.

The six selected AAST words were recorded in Solingen, Germany with a high-quality microphone, a Sennheiser model E914 and digital recording equipment. A female speaker with clear, natural pronunciation was used for the recordings. During the recording session, the speaker was asked to read out the list of 6 words twice. The recordings that best represented each of the 6 stimulus words were later chosen and saved. After the rating process, the intensity of each word was edited to yield the same intensity.

A calibration signal was also included, CCITT 1964. Speech-shaped noise was used as the masker. The same speaker was recorded whilst reading aloud from a book for 2 to 3 min. The speech was used to measure the long-term average speech spectrum. The noise was generated by superimposing the speech material, which produces noise with the same long-term spectrum as the speech material. This creates speech-shaped noise that best masks the speech material. Masking noise was continuous during the noise test. A high-frequency version was also developed for the Maltese AAST. Earlier versions of the high-frequency test differentiated words only by a single phoneme such as fricatives or voiceless plosives [34]. A whispered version of the quiet test was used as the high frequency version due to the lack of minimal pairs in the Maltese language.

### 2.2. Psychometric Curves for Maltese AAST

**Figure 2:**
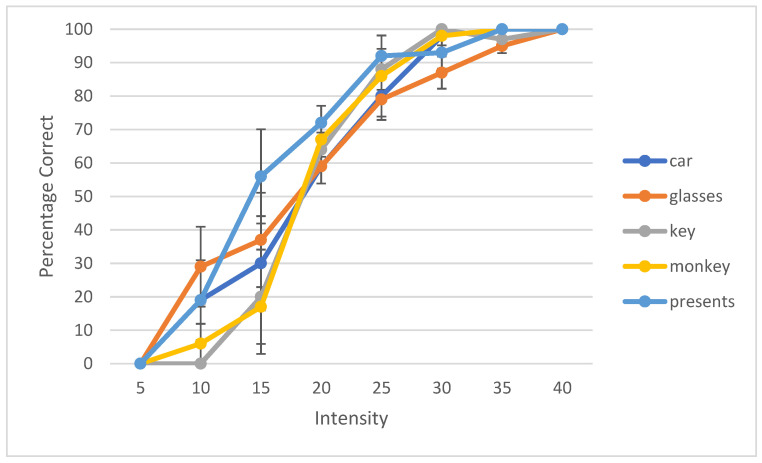
Psychometric properties for the Maltese AAST words in adults in quiet.

**Figure 3:**
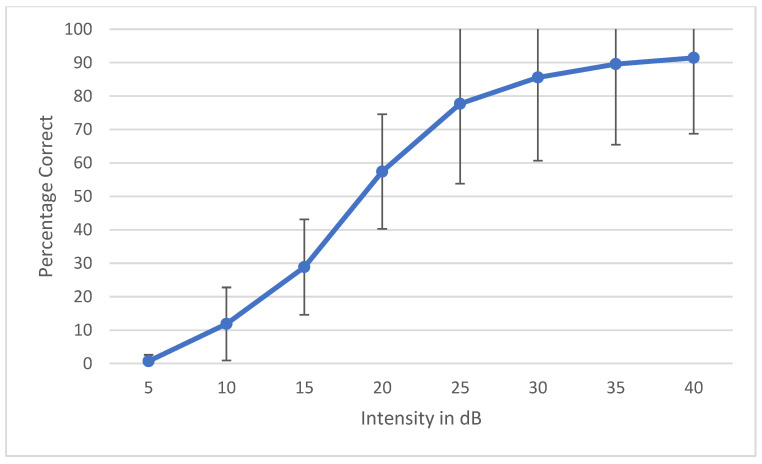
Average psychometric properties for the Maltese AAST words in adults in quiet.

**Figure 4:**
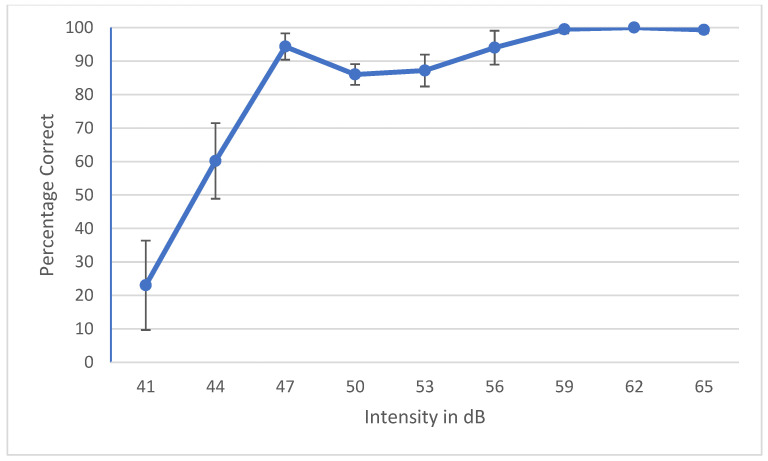
Average psychometric properties for the Maltese AAST words in adults in noise.

**Figure 5:**
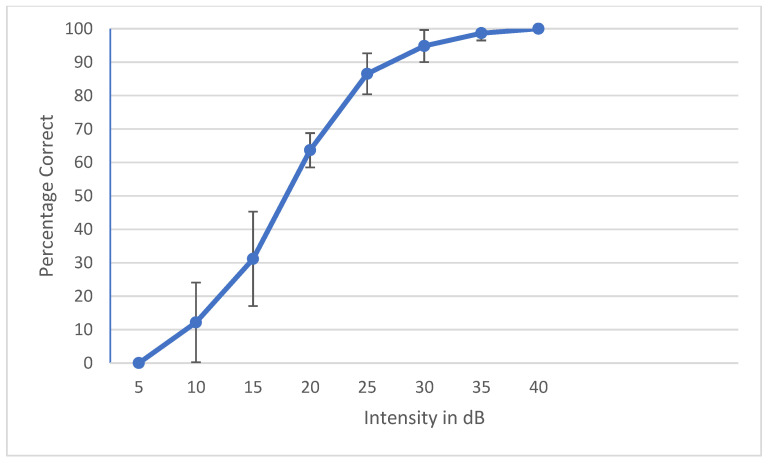
Average psychometric properties for the Maltese AAST words in adults in high frequency.

During the development of a speech audiometry test, one has to ensure that all the words are homogeneously audible. Currently, digital technology is widely used to make words equally audible, and hence, result in similar SRT results across words. Young et al. determined that the slope of each individual word should be ±1 SD of the mean to be considered homogenous [35]. Spondee words in English tend to have a steep slope due to their high audibility [36,37,38]. Research shows that the SRT materials developed in other languages have slopes of a psychometric performance-intensity function on trisyllabic words as steep as the slopes for English spondees [39]. To check the internal balancing of the 6 words, the AAST was tested on 30 adult normal hearing individuals in quiet, noise and high frequency. The results were plotted as psychometric curves, showing the relationship between the intensity sound level in the dB SPL unit on the horizontal axis against the percentage of the correct answers of adults on the vertical axis. Figure 2 shows the psychometric curve of all the six words in quiet. This confirms that the intelligibility degree of the 6 words is close and that words are not significantly easier or more difficult than each other. Figure 3, Figure 4 and Figure 5 show the average psychometric curves for the Maltese AAST words in quiet, noise and high frequency, respectively. The steepness of the slope of the AAST is 6.6%/dB in quiet, 14.3%/dB in noise and 6%/dB in high frequency.

### 2.3. Participants

In all, 248 listeners aged between 4 and 30 took part in the study on normative values. Participants included 208 children (aged 4, 5, 6, 7 and 10 years old, respectively) and 40 adults (18–30 years). The children were recruited from government and church schools. Informed consent was received from parents. The study was approved by the local university research ethics committee, UREC (Ref. No. UREC-DP1801016EXT). Data collection was conducted by Dr. Pauline Miggiani between March 2019 and March 2020. Children with medical conditions including hearing loss, multiple learning disabilities and syndromes were not included in the study. Non-Maltese-speaking children were also ruled out since the test material was delivered in the Maltese language. Data from several children had to be excluded because of incomplete measurements. These were either due to insufficient compliance or because the task was misunderstood. All 208 children had audiometric normal hearing (≤20 dbHL) at all frequencies tested (500 Hz, 1 kHz, 2 kHz, 4 kHz). Table 1 shows the gender distribution and the mean Pure-Tone Average for every age group.

### 2.4. Test Equipment

The tests were conducted at the paediatric audiology department of the local acute hospital or in a quiet room at the schools. At school, care was taken to ensure that the ambient noise did not exceed 40 dB(A). All testing was conducted using a Sennheiser HD 280 Pro closed circumaural headphone with a high ambient noise attenuation (<32 dB). The test words were presented through BELLS software running on a Microsoft Windows 10 touchscreen laptop for storing responses. The audio signal to the headphone was delivered through an external NuForce uDAC-2 asynchronous 24-bit, a USB Digital-to-Analog Converter headphone amplifier.

### 2.5. Procedure

All three versions, quiet, noise and high frequency, were carried out during the same session in the mentioned order, followed by Pure-Tone Audiometry. The order of presentation was counterbalanced by having testing starting on the right side in half of the children and vice versa. Frequent breaks were given in between tests to prevent fatigue, especially in the younger children. The data collection was conducted by the researcher.

All the participants were instructed at the beginning of the session. The AAST is a picture-pointing task on a touchscreen, and the test is initiated by pressing the start button in the centre of the screen. The participants hear the test words through the headphones. Before starting, the tester also ensured that the headphones adequately covered the listener’s ears. Once one of the test words is uttered, the participant needs to choose the presented item from a choice of 6 pictures on the screen, or to indicate that the item was not heard or not understood by pressing a question mark “?” button at the centre of the screen. Before starting the speech test, the children were made familiar with the test material to ensure that the test items were part of the child’s receptive vocabulary and that the images could be identified.

All participants were given the same verbal instructions to orient them to the nature of the task, to specify their mode of response, to indicate that the test material was speech and to stress the need for the child to respond at faint listening levels [1].

**Figure 6:**
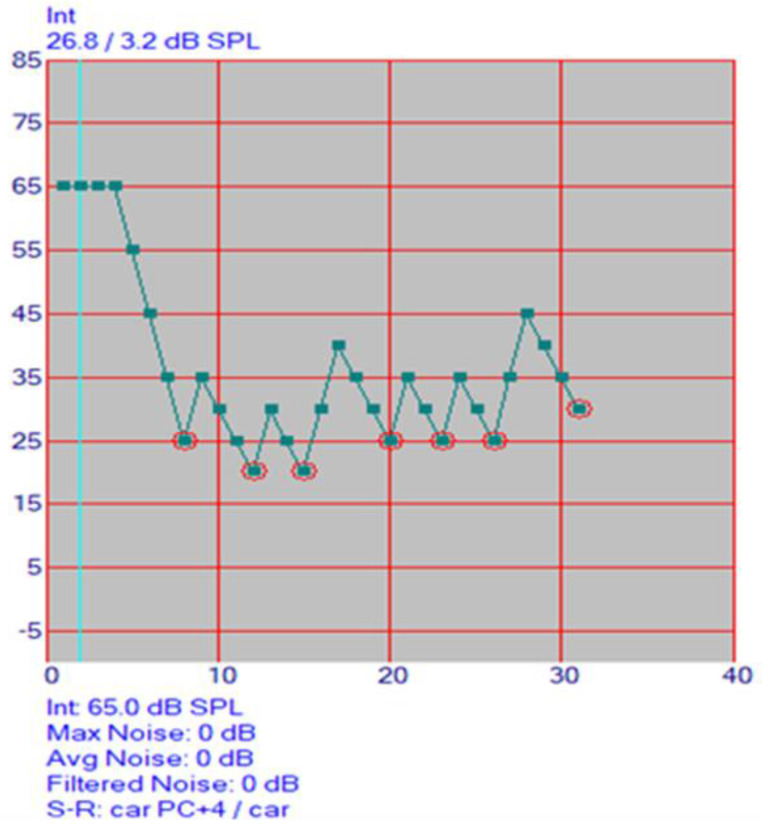
AAST result interface.

The presentation of the stimuli, the processing of the responses and the analysis were automatically carried out by the AAST program. At the beginning of the test, the level decreases in 10 dB-steps beginning from 60 dB until the first reversal occurs (incorrect or “?” input). After every correct answer, the next word is presented with 5 dB SPL lower volume. After every wrong answer, the volume is turned up by 10 dB SPL. In noise, the initial SNR is +5 dB, with the noise level at 65 dB SPL and start level of speech at 70 dB SPL. After every correct answer, the next word is presented with 3 dB SPL lower volume. After every wrong answer, the volume is turned up by 6 dB SPL. This up-down method adaptively determines the SRT in a quick and efficient manner. Figure 6 displays the audiogram proceeding. The SRT result is calculated as the mean of the presentation levels. The program stops automatically after seven incorrect answers.

During the measurements, the response time was also determined for each response as the time from the word offset to the onset of the touchscreen response. The responses were recorded and time-logged automatically by the software. The intensity of the stimulus word as well as the number of correct and incorrect answers was automatically saved by the AAST software.

## 3. Results

The aim of this study was to develop and norm a Maltese version of the AAST. The analysis of the scores was carried out using SPSS for Windows v.21 software and Microsoft Office Excel 2016.

The main factors that were tested were as follows:

- SRT values according to age and test setting (quiet, noise and high)
- Correlation between age and SRT
- Correlation between SRT in quiet and high frequency
- Test-retest reliability
- Correct responses according to age and test setting (quiet, noise and high)
- Response time according to age and test setting (quiet, noise and high)
- Reversal points

### 3.1. Normative Values

Scores were obtained from 248 Maltese-speaking participants. Non-parametric tests were used to analyse the data. The Shapiro–Wilk Test determined that the SRT distribution was non-normal in quiet, (W(493) = 0.935, *p* = 0.000), noise (W(496) = 0.944, *p* = 0.000) and high frequency (W(493) = 0.935, *p* = 0.000).

To determine the normative values of the SRT in the Maltese AAST, the participants’ SRTs were averaged for each age group. The mean SRTs among the age groups were compared to observe the differences in the SRTs as age increases. As displayed in Table 2, the median SRT threshold in all three test settings (quiet, noise and high frequency) decreases as the age increases. The interquartile range also decreases as age increases, indicating that the values are less spread out over a wider range. Large inter-individual variances of SRTs in young children are also evident. The last column displays the interquartile range between the 25th and 75th percentiles in what that age group would score. For instance, 4-year-olds scoring between f24 and 35.8 dB SPL in quiet would be considered to be within the norm.

**Figure 7:**
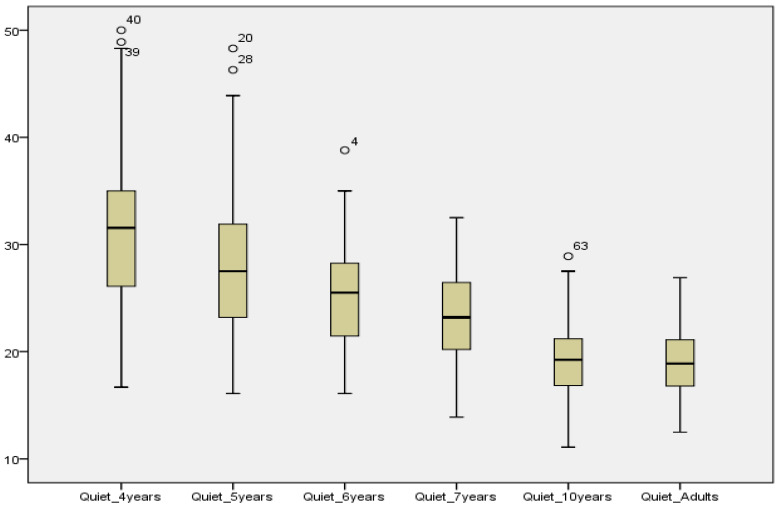
Comparison of mean SRT values in quiet across age groups.

**Figure 8:**
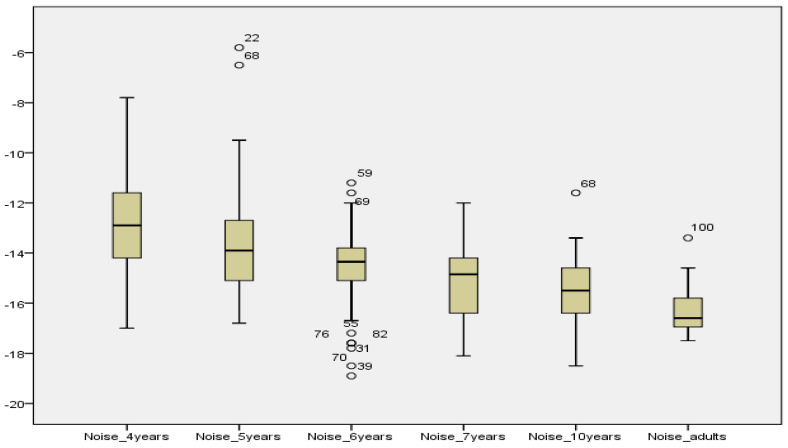
Comparison of mean SRT values in noise across age groups.

**Figure 9:**
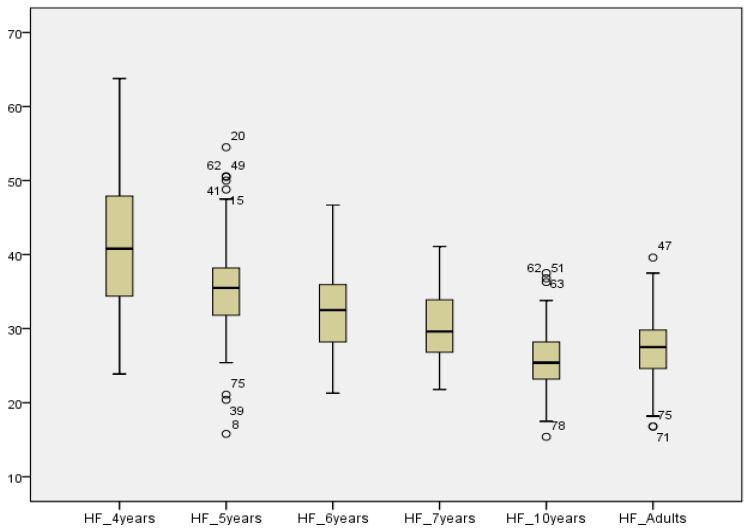
Comparison of mean SRT values in high frequency across age groups.

Kruskal–Wallis tests were used to compare AAST scores according to age. In all three test settings (quiet, noise and high frequency), the SRT decreased significantly between 4-year-olds and older children as shown in Table 3. In quiet and high frequency, no significant difference was reported between SRT scores in 10-year-olds and adults, showing that children at age 10 years had the same thresholds as in adults. See Figure 7, Figure 8 and Figure 9.

**Figure 10:**
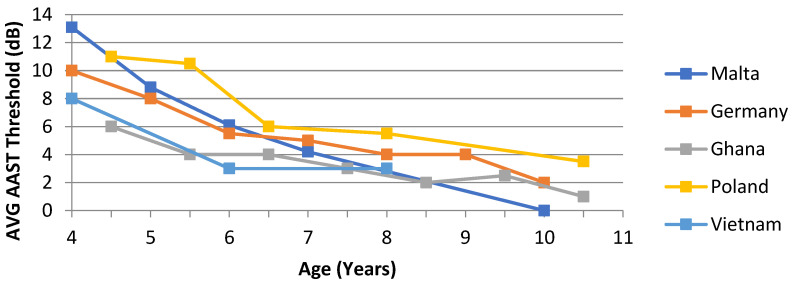
Age-related norm values in AAST in quiet across languages in comparison to the Maltese version. N.B. (Threshold plotted in *y* axis is the mean subtracted by 20dB; for example, in 6-year-old children, the average threshold is 25.3 dB SPL, and after correction with 20 dB, the THR is 5.3 dB HL).

In quiet, an age-dependent threshold difference of about 13 dB between younger and older children was observed. These findings are also plotted against other AAST versions for comparison. See Figure 10.

### 3.2. Correlation

#### 3.2.1. Correlation between Age and SRT

The correlations between the SRTs and age in the children’s groups were estimated by a slope value of 2.2 dB per year in quiet, 2.6 dB in high frequency and 0.5 dB in noise, respectively. There was an average difference of about 8dB SPL between SRT values in quiet and those in high frequency.

#### 3.2.2. Correlation between SRT in Quiet and High Frequency

A Spearman’s rank-order correlation was run to determine the relationship between the SRT values of listeners in quiet and high frequency settings. A strong positive correlation was reported in 4-year-olds (r (84) = 0.93, *p* = 0.000) and 7-year-olds (r (104) = 0.61, *p* = 0.000). A moderate correlation was reported in 5-year-olds (r (78) = 0.38, *p* = 0.000) and 10-year-olds (r (78) = 0.40, *p* = 0.000). The correlation was not significant in 6-year-olds (r (78) = 0.399, *p* =0.000) or adults (r (78) = 0.141, *p* = 0.213).

### 3.3. Test-Retest Reliability

Ten participants were retested a week apart to determine test-retest reliability of the Maltese version of the AAST in all three settings (quiet, noise and high frequency). Paired sample t-tests showed that there was no significant difference between the initial and second retest in the quiet (M = 16.89; SD = 4.67; M = 15.89; SD = 2.66; t(7) = 0.466, *p*= 0.656), noise (M = −16.69, SD = 1.07; M = −17.01, SD = 1.09; t(7) = 0.629, *p* = 0.549) or high frequency setting (M = 23.23; SD = 5.52; M = 21.61, SD = 2.49; t(7) = 0.591, *p* = 0.573.

### 3.4. Analysis of Correct Response

The number of correct responses was analysed according to the respective age groups. The crosstab results in Table 3 show that as age increases, the percentage of correct answers also increases. The chi-square test confirms that the difference is statistically significant, X^2^ (5, N = 248) = 58.936, *p* = 0.000), with a mean rank response time of 24,596 ms for 4-year-olds, 24,106 ms for 5-year-olds, 24,240 ms for 6-year-olds, 22,944 ms for 7-year-olds, 20,281 ms for 10-year-olds and 15,554 ms for adults. The number of correct responses was also analysed according to the test setting they were carried out in. Table 4 shows that the most correct answers are in quiet, followed by high frequency and least in noise. As seen in the chi-square tests in Table 3, this was statistically significant across all ages except in adults (*p* > 0.05). In adults, there was no statistically significant difference in correct answers in different settings.

### 3.5. Analysis of Response Time

In this research, response time was taken as the time from the word offset to the onset of the touchscreen response. The mean response time was 1.96 s with a minimum of −19.73 s and a maximum of 27.55 s. The negative response time was probably due to children pressing the picture before the stimulus word ends, questioning whether the child guessed rather than listened attentively.

An independent samples Kruskal–Wallis test showed that there was a statistically significant difference in the distribution of response times across the age categories (H = (2) = 13.577, *p* = 0.001). This suggests that response time decreases as age increases. Post hoc tests were conducted to evaluate pairwise differences among the 6 groups. There was a significant difference in adults (H (2) = 22.2, *p* = 0.000), 10-year-olds (H (2) = 37.3, *p* = 0.000), 7-year-olds (H (2) = 0.118, *p* = 0.003) and 4-year-olds (H (2) = 4.3, *p* = 0.117). No significant difference was reported between 5-year-olds (H (2) = 1.19, *p* = 0.552) and 6-year-olds (H (2) = 0.464, *p* = 0.537).

Post hoc pairwise comparisons were carried out. In adults, a significant difference was reported between quiet and high (*p* = 0.001) settings and quiet and noise (*p* = 0.000), whilst no significant difference was reported between noise and high (*p* = 0.436). In adults, the median response time was 1550 ms in quiet and high frequency and 1600 ms in noise. In 10-year-olds, a significant difference was reported between all settings; quiet and high (*p* = 0.002) settings and quiet and noise (*p* = 0.050) and noise and high (*p* = 0.000). In adults, the median response time was 1700 ms in high frequency, 1750 ms in quiet and 1700 ms in noise. In 7-year-olds, a significant difference was reported between noise and high (*p* = 0.001) settings and quiet and noise (*p* = 0.050), whilst no significant difference was reported between quiet and high (*p* = 0.129). In 7-year-olds, the median response time in quiet and high frequency was 1850 ms and 1900 ms in noise.

A Mann–Whitney U test showed that there was a significant difference (U = 175,377,990.0, *p* = 0.000) between the response times for correct answers when compared to the response times for wrong answers. The median response time for correct answers was 1800 ms and 2000 ms for wrong answers, suggesting that the response time was shorter when an answer was correct.

### 3.6. Analysis of Reversal Points

A reversal point in AAST testing is the last threshold in a series of descending thresholds at which the stimulus is correct. During this study, the software was set to stop at seven reversal points. An average was taken for all three test settings across each age group. In adults, in quiet, the average threshold at one reversal was 20.1 dB SPL and 19.3 dB SPL at seven reversals. In high frequency, the threshold at one reversal was 29.4 dB SPL and 27.8 dB SPL at seven reversals. In noise, the threshold at one reversal was −16.7 dB SNR and −16 dB SNR at seven reversals.

Independent samples of Kruskal–Wallis tests showed that there was no statistically significant difference between reversal points across age groups in quiet. See Table 5. This means that seven reversal points are more than enough to measure the SRT thresholds during the AAST. However, there was a significant difference in noise across all age groups except in 10-year-olds. Pairwise comparisons were carried out to investigate at which point the difference was statistically significant. In 4- and 6-year-olds, three reversal points were needed in noise, whilst only two reversal points were observed to be necessary in 5-, 7-, 10-year-olds and adults for running the AAST in noise.

## 4. Discussion

The purpose of this study was to develop and provide normative data for the AAST in the Maltese population. The aims were to determine the norms in quiet, noise and high frequency in Maltese-speaking adults and children aged 4 years and older. The sample included 248 Maltese-speaking children and adults.

### 4.1. AAST Normative Values in Quiet

The SRT in quiet decreases significantly between 4-year-olds and 10-year-olds. This means that older children perform better than younger children. No significant difference was reported between SRT scores in quiet in 10-year-olds and adults, showing that Maltese children at age 10 years achieve the same thresholds as adults. This suggests that speech recognition skills mature as children grow older and stabilise at around 10 years of age. The correlation between the SRTs and age in the children’s groups was estimated by a slope value of 2.2 dB per year in quiet.

Mohammed [31] reported similar values in 5- to 7-year-olds in quiet (26.6 dB SPL) in the Arabic version. This may be due to the language similarity between Arabic and Maltese. On the other hand, Maltese thresholds are noted to be slightly better than the Vietnamese norm values [30] across all ages; the 21–30-year-old group (29.4 dB SPL), the 15–20-year-old group (30 dB SPL), 8-year-olds (31 dB SPL), 6-year-olds (31.8 dB SPL) and 4-year-olds (37.2 dB SPL). Nguyen attributed these high thresholds to the high level of background noise during testing.

An age-dependent norm threshold difference was observed in the Maltese version of the AAST. There was a mean 13 dB difference between 4-year-old children and 10-year-old children in the AAST in quiet. Children aged 10 years achieved average speech thresholds that were comparable to adult thresholds. This trend is very similar across other AAST language versions. Coninx [21,22] reported a 10 dB difference between younger and older children in the German version, very similar to the Polish one, with an 11 dB difference. Offei [29] and Nguyen [30], on the other hand, reported a slightly less difference of 7 dB and 8 dB in the Ghanaian and Vietnamese version, respectively. Based on these findings, it can be confirmed that listener’s age and their SRT in the AAST are also interdependent in the Maltese AAST.

In the Maltese version of the AAST, the steepness of the slope was 6.6%/dB in quiet. Nguyen (2017) reported a slope of 8.2%/dB for the Vietnamese version, whilst Offei [29] reported a slope of 10.2%/dB in quiet for the Ghanaian version. The Oldenburger Kinder Satztest [28] has slopes of 6–8%/dB in quiet. These slight differences across AAST versions and other speech tests could be attributed to the sample composition in the population under study.

A weak-to-moderate correlation was observed between SRT values in quiet and the participants’ PTA averages. This suggests that an increase in the participants’ PTA averages were only weakly to moderately correlated with an increase in SRT levels in quiet.

### 4.2. AAST Normative Values in Noise

The features of SRTs in noise were relatively similar to those in quiet. The mean SRTs in noise obtained by the younger children decreased significantly between 4-year-olds and adults. The correlation between the SRTs and age in the children’s groups was estimated by a slope value of 0.5 dB per year in noise. This close correlation was also reported by Nguyen [30] with a slope value of roughly −1 dB per year.

Regarding psychometric function, the Maltese version has a slope of 14.3%/dB in noise, very similar to the German AAST [21,22] with a slope of 14%/dB and to the Oldenburger Kinder Satztest [28] with slopes of 12–14%/dB. On the other hand, Nguyen [30] reported a slope of 8.4%/dB in noise. Whilst a small increase in SNR in the German and Maltese version of the AAST led to a large increase in intelligibility, the same SNR improvement led to a smaller perceptual improvement in speech intelligibility.

In comparison to Mohammed’s study [31], Arabic SRT thresholds in noise were somewhat similar to those of the Maltese version, with 5-year-olds having an average speech in noise threshold of −10.8 dB SNR, 6-year-olds having a mean of −11.53 dB SNR and 7-year-olds with a mean of −11.95 dB SNR. Similarly, Nguyen reported 4-year-olds having poor mean speech threshold values between −9.5 and −6.5 dB SNR. The mean SRT of adults in Vietnamese was approximately −14 dB SNR, which is also slightly below the Maltese mean SRT in noise. Overall, one can conclude that Maltese children and adults scored better than Arabic and Vietnamese participants in noise.

### 4.3. AAST Normative Values in High Frequency

The speech recognition thresholds in high frequency decrease significantly as the age of the children increases. Adults perform as well as 10-year-olds in the Maltese high-frequency version, similar to results obtained in the quiet setting. In addition, the correlation between the SRTs and age in the children’s groups is also very similar, with a value of 2.2 dB per year in quiet and 2.6 dB in high frequency. A moderate-to-strong correlation is reported between SRT values in quiet and high frequencies across age groups. An average difference of about 8dB SPL was reported in SRT values in quiet and in high frequency, confirming that the high-frequency version is more demanding on the listener.

Whispered speech is acoustically characterised by the lack of vocal folds’ vibration. Whispering affects only voiced sounds, such as vowels, which are produced by forcing air through a constricted opening between the vocal folds in the larynx. In fact, voiceless fricatives are not affected at all by whispering. Whispered speech tends to be higher in frequency than the corresponding voiced speech. According to Vestergaard and Patterson [34], whispering removes the natural temporal fine structure of voiced speech, which may affect the redundancy of speech information. This would explain the higher thresholds obtained in this study.

Some conclusions can be drawn from the speech recognition performance of Maltese listeners on the Maltese AAST. The results of this study indicate that the SRT values are age dependent, as a significant improvement in threshold is observed as children grow older. This was observed in the three test settings: quiet, noise and high frequency. In quiet and high frequency, 10-year-olds obtained a SRT which was comparable to that of adults, suggesting that speech recognition skills are mature in normal-hearing older children. In quiet, a 13 dB difference was noted between 4-year-old and 10-year-old children. This age-related difference could be due to the fact that speech perception relies on several cognitive and linguistic factors, including selective attention, short-term memory and lexical knowledge, which develop as children get older. Overall, the children were attentive in the test situation since it was displayed on a tablet PC, confirming that the AAST is appropriate for use in young children.

### 4.4. Test-Retest Reliability

Test-retest reliability of the Maltese version of the AAST was also confirmed on a small sample of participants. There was no significant difference between the initial and second retest in the quiet, noise or high frequency setting. These measurements, obtained in one sitting, were both representative and stable over time, preventing any age-related changes in performance or learning effects. Previous AAST studies by Mohammed [26] and Nguyen [25] also show that there are no learning effects in the Arabic and Vietnamese versions, respectively. Offei [24], on the other hand, reported some learning effects in children but not in adults. Nguyen [25] stated that although there was no statistically significant difference in his study, the improvements in SRTs (1–2.5 dB) may affect the clinical findings to some degree.

### 4.5. Correct Answers

The findings in this study show that as age increases, the percentage of correct answers also increases significantly. Children scored more correct answers in quiet, followed by high frequency and least in noise. However, adults scored as well in quiet as in the other two settings. These findings suggest that speech recognition abilities of children keep on developing throughout childhood, and their ability to decode degraded signals such as speech in noise and high frequency improves accordingly with age.

### 4.6. Response Time

Response time is also an interesting factor to consider in the analysis of automatic adaptive speech tests. Results from this study confirm that response time is also dependent on the listener’s age. As children get older, response time decreases significantly, possibly in relation to the maturity of attention and concentration skills, which are still developing in very young children.

Response times across the different test settings (quiet, noise or high frequency) were also evaluated in this study. While there was no significant difference in response time between the different test settings in younger children (4-, 5- and 6-year-olds), a significant difference was observed in older children and adults. In 7-year-olds and 10-year-olds, response time was significantly longest in noise and shortest in high frequency. In adults, the longest response time was also in noise, followed by high frequency and shortest in quiet. Overall, it can be concluded that as children grow older, listening in noise remains a challenge, as evidenced by the longer response times. This finding highlights the importance of using response time in speech tasks as a measure of the cognitive load in speech recognition. Similar findings have been reported across studies [11,12,40,41]. As listening conditions become more degraded, listening effort also increases, as evidenced by an increase in response time.

### 4.7. Reversal Points

During this study, the software was set to automatically stop at seven reversal points. In general, the accuracy and reliability of the threshold estimate may be improved by increasing the number of reversals. However, time may be a limiting factor when testing children, as they may become restless or inattentive. A child with a short attention span may respond inconsistently and only at supra-threshold levels, leading to elevated SRT levels. Children may be unable to complete the full number of reversals, and thus the relationship between SRT and reversal points was evaluated in this study across different age groups and test settings (quiet, noise and high frequency).

In this study, there was no statistically significant difference between reversal points across any age group in quiet and high frequency settings. This means that seven reversal points are more than enough to measure the SRT thresholds during the AAST. In noise, a significant difference was observed across all age groups except in 10-year-olds. Detailed analysis showed that on average two or three reversal points were necessary to maintain stable AAST thresholds across age groups in noise. This is an important finding for clinicians who would like to use the Maltese version of the AAST for screening purposes. It allows the possibility to screen more children in a shorter time and enable reliable results in children who have a short attention span.

## 5. Summary

In conclusion, the findings of this study confirm the validity and reliability of the Maltese version of the AAST in establishing the SRT in Maltese-speaking children and adults. The results of the study suggest that adaptive speech tests such as the AAST can be helpful in quantifying speech recognition in Maltese-speaking listeners. Especially for young children, there are very few subjective methods that can be used reliably and at the same time are appropriate for children. The findings from this study serve as an initial step in exploring measures that evaluate speech perception in the Maltese Islands through the collection of normative data. Results from normal populations will allow the specification of norms which can be used as a reference point when testing patients clinically.

The AAST may also serve as a screening and assessment tool for the early identification of hearing loss in the Maltese population, potentially filling in the gap between the diagnosis/screening between birth and school. The Maltese version of the AAST may potentially be used as a school entry screening tool in Maltese-speaking school age children. School entry hearing screening would have an important role in early identification. The Maltese AAST provides objective information on a child’s speech perception development, thus enabling comparisons between children of varying ages. Normative data would enable better identification and management of children with hearing impairment.

The findings show that the AAST is a reliable and valid screening tool which will improve the quality of speech audiometry in Malta. The advantages of using the AAST in young children may also be valid for the elderly, it being a short, closed set task that has less cognitive demands. More research in the area is needed to examine how hearing screening in the older population may be carried out using the AAST.

## Figures and Tables

**Table 1:** Number of participants for every age group and Mean PTA.

Age (Years)	Number of Participants	Mean PTA (dBHL)	SD
4	40 (16 males, 24 females)	12.6	4.72
5	40 (24 males, 16 females)	10.3	4.24
6	38 (23 males, 15 females)	8.4	2.86
7	50 (22 males, 28 females)	7.3	3.29
10	40 (15 males, 25 females)	5.4	2.80
Adults (18–30)	40 (16 males, 24 females)	5.2	2.57

**Table 2:** Median and Interquartile Range Percentiles in Quiet, Noise and High Frequency across age groups.

	Quiet			Noise			High Frequency		
Age	Median	IR	25th–75th %tile	Median	IR	25th–75th %tile	Median	IR	25th–75th %tile
4 years	33.1	9.7	35.8	−12.9	2.9	−11.4	40	13.6	47.5
5 years	27.5	8.7	31.9	−14.0	2.2	−12.9	35.6	6.4	38.2
6 years	25.6	6.5	28.3	−14.6	1.3	−13.8	33.2	7.4	36.3
7 years	23.2	6.8	26.8	−15.1	2.2	−14.2	29.6	7.1	33.9
10 years	18.9	4.3	21.1	−15.5	1.8	−14.6	25.4	5.0	31.1
Adults	18.9	4.3	21.1	−16.6	1.3	−15.8	27.5	5.4	30

**Table 3:** Pairwise comparisons of AAST scores in quiet, noise and high across the different age groups.

Age Groups	Quiet	Noise	High
4–5-year-olds	0.005	0.003	0.001
5–6-year-olds	0.009	0.001	0.003
6–7-year-olds	0.023	0.019	0.013
7–10-year-olds	0.000	0.005	0.000
10–Adults (18–30)	0.959	0.000	0.237

**Table 4:** Percentage of correct responses for AAST in quiet, noise and high frequency settings according to age.

Age	Overall Correct	Quiet	Noise	High Frequency	Chi-Square Value	df	*p*-Value
4	68.2%	70.4%	66.5%	67.6%	8.887	2	0.012
5	69.9%	71.3%	67.9%	70.2%	6.498	2	0.039
6	70.9%	72.6%	68.7%	71.1%	8.066	2	0.018
7	71.4%	73.2%	69.1%	71.7%	12.273	2	0.002
10	72.4%	74.1%	70.2%	72.7%	9.000	2	0.011
Adults	73.4%	74.3%	73.4%	72.6%	1.815	2	0.404

**Table 5:** Independent samples Kruskal–Wallis Tests for Reversal Points across quiet, noise and high frequency settings.

Age Groups	Quiet			High			Noise			Reversal Points Needed
	H	df	*p* Value	H	df	*p* Value	H	df	*p* Value	
4-year-olds	3.39	6	0.759	2.9	6	0.815	39.6	6	0.000	3
5–year-olds	0.635	6	0.996	2.7	6	0.851	36.4	6	0.000	2
6–year-olds	7.5	6	0.279	0.120	6	1.000	28.3	6	0.000	3
7-year-olds	3.5	6	0.748	1.6	6	0.959	15.7	6	0.000	2
10-year-olds	0.571	6	0.997	5.3	6	0.504	3.8	6	0.707	1
Adults	0.983	6	0.986	3.1	6	0.794	56.0	6	0.000	2

## Data Availability

Not applicable.
